# Isotopic turnover rates and diet-tissue discrimination depend on feeding habits of freshwater snails

**DOI:** 10.1371/journal.pone.0199713

**Published:** 2018-07-05

**Authors:** Chen-Hua Li, James D. Roth, Jillian T. Detwiler

**Affiliations:** 1 Department of Biological Sciences, University of Manitoba, Winnipeg, Manitoba, Canada; 2 Department of Ecosystem and Public Health, Faculty of Veterinary Medicine, University of Calgary, Calgary, Alberta, Canada; Waseda University, JAPAN

## Abstract

Estimates of animal diets and trophic structure using stable isotope analysis are strongly affected by diet-tissue discrimination and tissue turnover rates, yet these factors are often unknown for consumers because they must be measured using controlled-feeding studies. Furthermore, these parameters may be influenced by diet quality, growth, and other factors. We measured the effect of dietary protein content on diet-tissue discrimination and tissue turnover in three freshwater snail species. We fed lettuce to individually housed snails (n = 450 per species) for ten weeks, then half were switched to a high-protein diet. Isotopic values of muscle and gonad tissue were assessed at 48 and 80 days post-diet change. Snail discrimination factors varied by diet (low-protein > high-protein) and usually differed among species for both N and C, although species had similar carbon discrimination when fed the low-protein diet. Carbon turnover rates were similar among species for a given tissue type, but nitrogen turnover varied more among species. In addition, diet affected growth of species differently; some species grew larger on high-protein (*H*. *trivolvis*) while others grew larger on low-protein diet (*Lymnaea* spp.). These differences among species in growth influenced turnover rates, which were faster in the species with the highest growth rate following the diet switch from low to high-protein. Thus, growth is one of the main processes that affects tissue turnover, but growth and feeding preference did not affect diet-tissue discrimination, which was greater on low-protein than high-protein diets for all species regardless of growth performance. These results suggest that diet might influence two key parameters of stable isotope analysis differently.

## Introduction

Stable isotope ratios are commonly used to reconstruct consumers’ diets and determine trophic structure in ecological communities [1, 2] but the accuracy of diet estimates from this approach is influenced by our understanding of diet-tissue discrimination (also called “trophic discrimination”, “trophic enrichment” or “fractionation”) and tissue turnover rates [3, 4]. Diet-tissue discrimination factors (DTDFs, the difference between the isotopic value of the consumer tissue and the diet) and tissue turnover rates (how quickly the tissue shows the isotopic signature of the diet) may be affected by several characteristics, including species [5], tissue type [5–7], and diet quality [8]. However, these parameters have been measured in only a few species using controlled-feeding studies.

Stable isotope analysis can be used to reconstruct consumers’ diets because the isotopic composition of animal tissue reflects their diet’s isotopic composition for a certain period of time. Stable isotope ratios are shown in δ notation in parts per mil (‰): δ X = (R_sample_ / R_standard_− 1)*1000, where X is the heavier isotope (^13^C or ^15^N) and R is the ratio of heavier to lighter isotopes (^13^C/^12^C or ^15^N/^14^N) in the sample tissue and in the national standard reference material (Vienna Pee Dee Belemnite (VPDB) for carbon and atmospheric N_2_ for nitrogen). δ^13^C can provide information on primary producers because C_3_ and C_4_ plants have distinctive δ^13^C values [9, 10], whereas δ^15^N can estimate the trophic position of consumers because it changes predictably with trophic level [1, 2]. Integrating δ^13^C and δ^15^N values into Bayesian stable isotope mixing models can reconstruct complex diets (three or more prey/plant items) for wildlife populations over weeks to years depending upon the species and the tissue being analyzed [2, 11, 12]. Stable isotope mixing models must include values for DTDF (Δ^13^C = δ^13^C_tissue_ - δ^13^C_diet_ and Δ^15^N = δ^15^N_tissue_ - δ^15^N_diet_). In most cases, DTDF has not been directly determined for a particular consumer with controlled feeding studies and instead values from a related species (ecologically or phylogenetically) are used.

The amount of protein in the diet influences DTDFs because of differences in the type and amount of amino acids in animal and plant tissues [13–15]. For omnivores, protein from animal tissues can be broken down into more amino acids than plant-based proteins [13]. Moreover, animal tissues generally have higher protein content than plant tissues because animal cell walls are mainly protein, whereas plant cell walls consist of mainly carbohydrate [14]. These characteristics suggest that for omnivores, diet quality increases with increasing proportions of animal protein in the diet [8]. For herbivores, animal-derived protein is not as suitable and they must derive more amino acids from other dietary sources (i.e., carbohydrates) compared to carnivores or omnivores. Because protein is generally enriched in ^13^C up to 3‰ relative to carbohydrates, herbivores have higher DTDFs than carnivores [16].

Turnover rate, often measured as half-life or the time it takes for 50% of the stable isotopes in the tissue to be replaced by the stable isotopes in the diet, is mainly affected by metabolic rate [6, 17–19]. Metabolism among tissues can differ because of the rate at which old tissues are replaced, especially for protein metabolism. More metabolically active tissues reflect the diet within a few weeks, but tissues with lower metabolic rates reflect the diet from the previous few months [6, 19]. Many animals are still growing when they are incorporating their diet into their tissues, so measurements of turnover rates may also be affected by growth, which involves new tissue synthesis (e.g. somatic growth).

Controlled-feeding studies can be challenging or impossible depending upon the consumer species of interest because known diets must be fed to consumers in a consistent environment for a sufficient time (e.g. [5–7, 20]). As a result, turnover rates and discrimination factors from phylogenetically or trophically similar species are often applied to species in which these values have not been directly measured (e.g. [21–23]). However, small differences in DTDFs can lead to large differences in diet estimates from stable isotope ratios [24, 25]. For example, Bond and Diamond (2011) used DTDFs published for different piscivorous birds to estimate the proportion of krill in a tern’s (*Sterna hirundo*) diet and depending upon the DTDF the results varied from 10.8% to 90.7%. Consequently, the use of inaccurate parameters in stable isotope mixing models could influence conservation and management decisions [25].

More laboratory-based feeding experiments are needed to accurately estimate DTDF and turnover rates [3, 26], especially for taxa, like invertebrates, that are particularly underrepresented [9]. A recent review of DTDFs included 93 published pairs of estimates for carbon and nitrogen from 44 species, which is staggeringly small considering the millions of invertebrate species [27]. Similarly, half-life has been determined for relatively few invertebrates. A review based their conclusions on 56 pairs of half-life for 16 invertebrate species [28]. In most cases, the whole organism was used to generate DTDFs and half-life for invertebrates (84/93 cases for DTDFs and 43/56 cases for turnover rate estimates); thus, tissue-specific estimates are more scarce. For the invertebrates whose DTDFs have been measured in the laboratory, only a third were aquatic species and five of those were freshwater species.

We focused on freshwater snails because they are ubiquitous in freshwater ecosystems and are primary consumers that alter the amount of detritus, algae, and periphyton (a complex mixture of algae, cyanobacteria, heterotrophic microbes, and detritus) in wetlands, streams, and lakes [28]. In addition, snails play a key role in disease transmission in freshwater ecosystems because they are hosts for many parasites including trematodes. Differences in diet among snail species could reflect microhabitat use, which could then be used to predict where hot spots of transmission to wildlife could occur. Moreover, knowing the DTDF and turnover rates for snails provides an opportunity to test how parasitism influences snail host diet.

The objective of this study was to determine DTDFs and turnover rates of carbon and nitrogen stable isotope ratios in muscle and gonad of three freshwater snail species (*Helisoma trivolvis*, *Lymnaea elodes*, and *Lymnaea stagnalis*) raised in the laboratory. We manipulated diet by switching from a low-protein to a high-protein diet because the high-protein diet had a higher isotopic value for both carbon and nitrogen compared to the low-protein diet. Differences in DTDFs and turnover rates among species were predicted because of trophic differences (*H*. *trivolvis* = generalist feeder; *L*. *elodes* = algae and carrion specialist; *L*. *stagnalis* = herbivore feeding primarily on macrophytes and algae) [29, 30]. We predicted that the herbivore (*L*. *stagnalis*) would have higher DTDFs compared to the omnivores (*H*. *trivolvis* and *L*. *elodes*). Due to the trophic differences among the species, we also predicted that diet quality would affect snail growth. We expected the generalist feeder *H*. *trivolvis* would grow larger on high-protein diet compared to low-protein diet, whereas the primarily plant-feeding *Lymnaea* spp. would grow larger on low-protein diet compared to high-protein diet. We predicted that snail gonad tissue would have lower DTDFs and faster turnover rates compared to muscle tissue. Gonads are more metabolically active since egg-laying activity starts around two months of age for all three species, and gonadal maturation and gonaduct functions are required for egg production [31–33]. By determining these parameters, we can more accurately estimate trophic positions in food webs involving aquatic snails and test how diet preference affects DTDF and turnover rates in wildlife diet reconstruction.

## Methods and materials

### Laboratory diets

We raised snails on a low-protein diet that consisted of well-rinsed green leaf lettuce purchased from a local grocery store. We also created a high-protein diet, based on a recipe by Sandland and Minchella [34], that consisted of 1 g BactoAgar (powder derived from algae), 1 g corn powder and 10 g Tetramin RichMix (40% protein, 5% fat, 2% fiber, 6% moisture, 1.3% phosphorous) mixed with 83 mL of boiled water. The corn powder, which is ^13^C-enriched, was added to ensure that the turnover of carbon was detectable. The mixture was poured into a tray (0.5 cm depth) and placed in a drying oven at 60°C for 12 hours to prevent bacterial growth.

### Snail sampling

For each species of snail, 450 hatchlings from laboratory breeding colonies (established from field-collected snails in southern Manitoba) were raised individually in separate 100 mL glass jars with 80 mL of well water. All snails were maintained in a room with a 12h:12h light/dark scheme. Each individual was fed green leaf lettuce *ad libitum* for ~10 weeks until shell length was approximately 10 mm (from apex to aperture, [35]). Then the snails were randomly assigned into a control or experimental group (n = 225 for each). Control snails continued their lettuce (low-protein) diet, while the experimental snails were switched to the high-protein diet. All snails were fed three times per week with a water change before feeding for the duration of the experiment. Snails from both groups were subsampled at 1, 2, 4, 8, 16, 32 and 48 days post-diet change for *H*. *trivolvis* and *L*. *elodes*. Additional subsampling for *L*. *stagnalis* included 64 and 80 days post diet-change because 48 days was not sufficient for *L*. *elodes* to achieve turnover (see Results). For each subsample, 30 snails from each group were randomly chosen, and the length of each individual was measured to the nearest 0.01 mm. Muscle (foot) and gonads from ten snails were each pooled into a sample to ensure that there was enough tissue for stable isotope analysis (three pooled replicates per subsample). Tissue samples were frozen at -20°C.

### Sample processing for stable isotope analysis

In addition to snail tissue, we sampled each head of lettuce (n = 4 for *H*. *trivolvis*, n = 5 for *L*. *elodes*, n = 3 for *L*. *stagnalis*) and each batch of the high-protein diet (n = 2 for each species) that snails were fed. Snail tissue samples and high-protein diet samples were freeze-dried for 48 hours, and then ground to a fine powder with a mortal and pestle. We extracted lipids from the snail and diet samples because lipids have different carbon isotopic composition than other components, which could lead to high variation in δ^13^C measurement for samples with different amounts of lipids [36]. Lipids were removed from samples with petroleum ether for 16 hours using a Soxhlet apparatus, and then oven dried at 60°C for 24 hours [37]. Low-protein (lettuce) samples were placed in a drying oven at 60°C for 48 hours and then ball-milled to a fine powder. The powdered samples were weighed (0.4–0.6 mg for snail tissue and high-protein diet; 2.5–3 mg for low-protein diet) and sent to the Chemical Tracers lab at the Great Lakes Institute of Environmental Research (GLIER), University of Windsor, Stratford, Ontario and processed with an Isotope Ratio Mass Spectrometer (Thermo Delta V) to determine carbon and nitrogen stable isotope ratios.

### Data analysis

General linear models were used to determine the influence of species, diet, time and tissue on δ^13^C and δ^15^N values, respectively. An additional general linear model was used to determine the effect of snail species, diet and time on size (shell length). Model selection began with consideration of a full model including all main effects, covariates, and interactions terms. Components were dropped if they were not significant (*P* > 0.05) and the Akaike information criterion (AIC) value was lower without the component in the model [38].

To model turnover of carbon and nitrogen isotopic values, we assumed an exponential pattern [6, 17] and fitted δ^13^C and δ^15^N values of each tissue type to equation:

$$\delta^{13}C\;or\;\delta^{15}N=\delta_{n}+(\delta_{0}-\delta_{n})e^{-\lambda t}$$ (1)

where *δ*_*n*_ represents the isotopic value at equilibrium, *δ*_*0*_ represents the δ value prior to the diet switch, *λ* is the turnover rate (%/day) derived from the model, and *t* is the time of feeding with the new diet (days). Half-life was calculated as ln(2)/λ, which is the time (days) it takes for 50% of the tissue to be replaced. We also calculated the time it takes for 99.99% of tissue to be replaced as ln(10000)/λ as recommended by Tieszen *et al*. [6].

Δ^13^C and Δ^15^N were calculated for each snail species (*H*. *trivolvis*, *L*. *elodes*, *L*. *stagnalis*) and tissue type (muscle and gonad) by comparing the mean diet isotopic value and *δ*_*n*_ in Eq 1 for snails fed the high-protein diet. For the low-protein diet, DTDFs were calculated by subtracting the mean isotopic value of the snail tissue in all sample days and the mean isotopic value of the diet. The standard deviation (SD) of DTDFs was calculated with the following equation:

$${SD}_{DTDF}=\sqrt{{SD}_{tissue}^{2}+{SD}_{diet}^{2}}$$

General linear models determined whether Δ^13^C and Δ^15^N values differed between the species, diet, and tissue types. Mean Δ^13^C and Δ^15^N values for lettuce-fed snails were calculated for each sample day using the three replicate samples because these snails had always been fed lettuce. For protein-fed snails, only values from the last sample day were used because tissue turnover plateaued at this point (day 47 for *L*. *elodes*, day 48 for *H*. *trivolvis*, and day 80 for *L*. *stagnalis*). Model selection was as described above. All statistics were performed in R version 3.2.5 [39] using packages MASS [40].

## Results

Diet, time and tissue type explained most of the variation in δ^13^C (r^2^ = 0.93) (Table 1). For the model of δ^15^N, all factors were significant (r^2^ = 0.86; Table 1).

**Table 1:** General linear model results for the effect of species, diet, time, tissue, and significant interactions on δ^13^C and δ^15^N values.

Factors	δ^13^C			δ^15^N		
	df	*F*	*P*	df	*F*	*P*
Species^a^	2, 236	0.22	0.806	2, 232	18.02	< 0.001
Diet^b^	1, 236	2475.91	< 0.001	1, 232	787.09	< 0.001
Time	1, 236	252.22	< 0.001	1, 232	20.27	< 0.001
Tissue^c^	1, 236	125.92	< 0.001	1, 232	180.71	< 0.001
Species × Diet	2, 236	16.00	< 0.001	2, 232	64.31	< 0.001
Species × Time	-	-	-	2, 232	27.22	< 0.001
Diet × Time	1, 236	435.09	< 0.001	1, 232	197.00	< 0.001
Diet × Tissue	1, 236	43.91	< 0.001	1, 232	16.05	< 0.001
Species × Diet × Time	-	-	-	2, 232	16.69	< 0.001

^*a*^*Helisoma trivolvis*, *Lymnaea elodes*, *Lymnaea stagnalis*

^*b*^High-protein diet, low-protein diet

^c^Muscle, gonad

### Turnover rates

**Fig 1:**
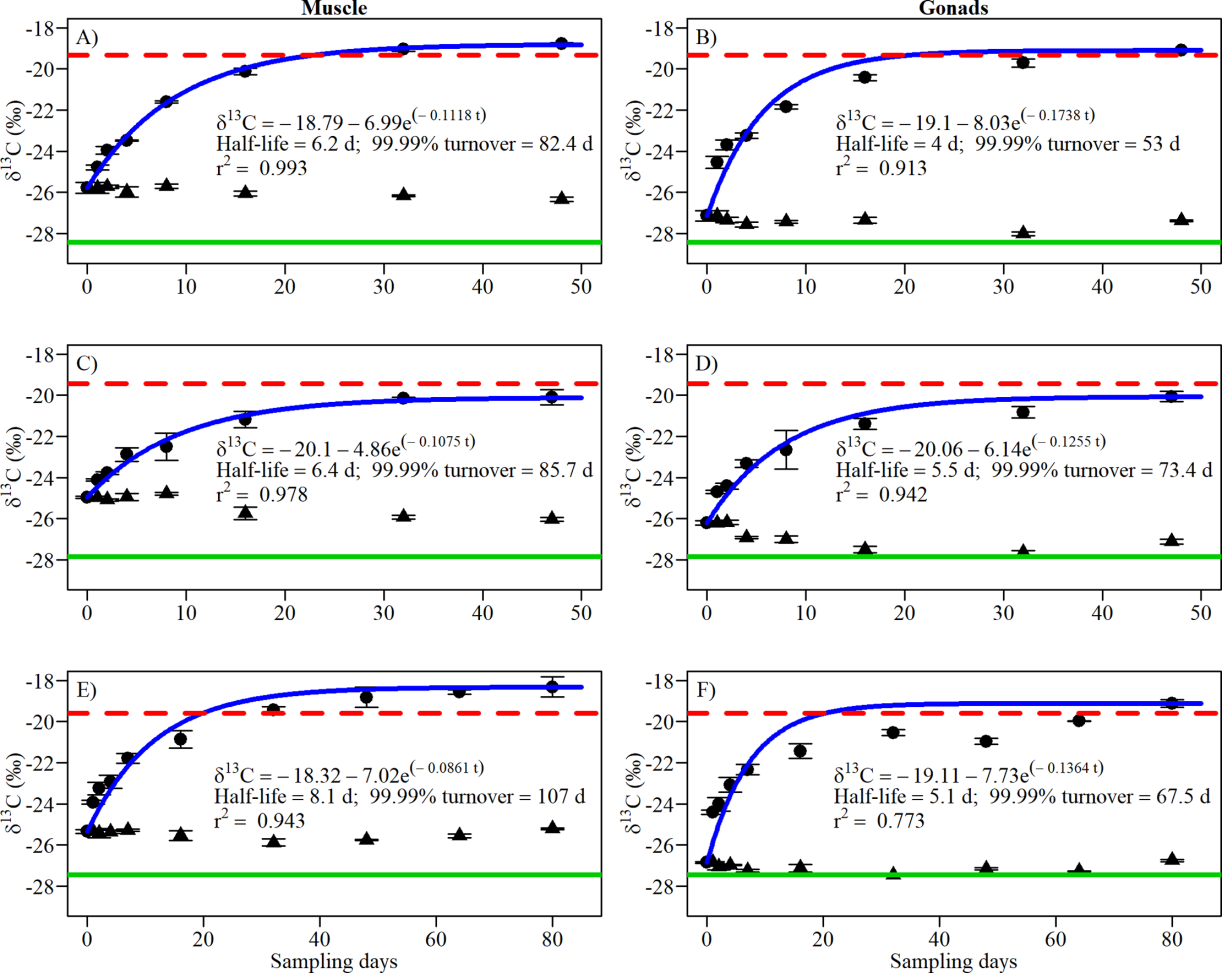
δ^13^C values of muscle (left) and gonad tissue (right) per sampling day (mean ± SE) for three species of freshwater snails fed low-protein (triangles) or high-protein diet (dots). (A–B) *Helisoma trivolvis*, (C–D) *Lymnaea elodes* and (E–F) *Lymnaea stagnalis*. For each graph, the blue curve is the best fit, the dotted red line represents the mean δ^13^C value of the high-protein diet (n = 2 for A–F), and the green line represents the mean δ^13^C value of the lettuce diet (n = 4 for A–B, 5 for C–D, 3 for E–F).

**Fig 2:**
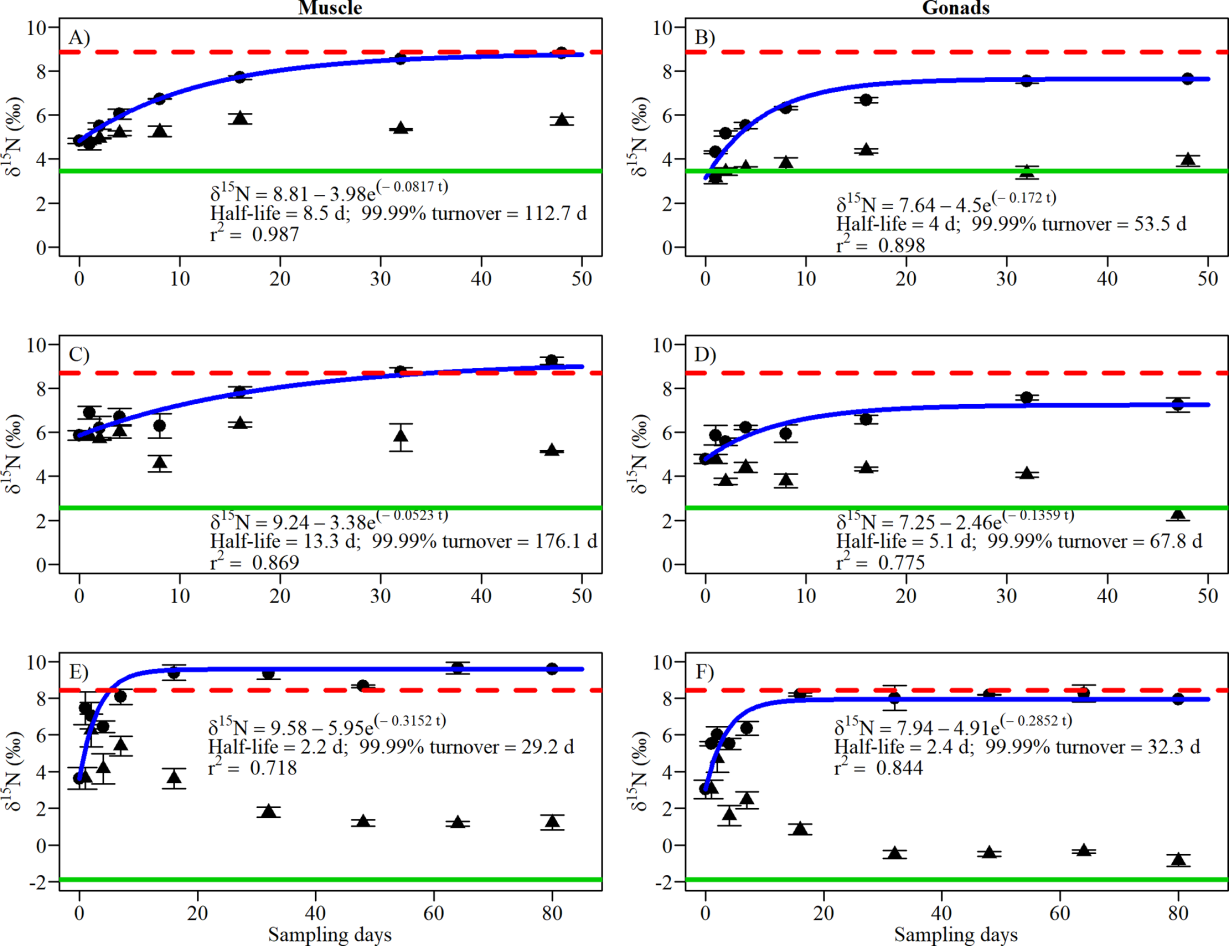
δ^15^N values of muscle (left) and gonad tissue (right) per sampling day (mean ± SE) for three species of freshwater snails fed low-protein (triangles) or high-protein diet (dots). (A–B) *Helisoma trivolvis*, (C–D) *Lymnaea elodes* and (E–F) *Lymnaea stagnalis*. For each graph, the blue curve is the best fit, the dotted red line represents the mean δ^15^N value of the high-protein diet (n = 2 for A–F), and the green line represents the mean δ^15^N value of the lettuce diet (n = 4 for A–B, 5 for C–D, 3 for E–F).

Snail stable isotope ratios began changing within days after switching the diet (Figs 1 and 2). Snail gonad tissue had faster turnover rates than muscle tissue for both carbon (half-life: 5–5.5 days vs. 6.1–8 days) and nitrogen (half-life: 2.4–5.1 days vs 2.2–13.3 days) isotope ratios. The half-life of δ^13^C for each tissue type was similar among species (max difference of 1.9 days for muscle, 1.5 days for gonad). Variation in half-life within tissue among species was greater for δ^15^N (max difference of 11.1 days for muscle, 2.7 days for gonad) compared to δ^13^C. The differences among species and tissue were more apparent with the 99.99% turnover time. In some cases, snail tissue achieved nearly complete turnover in approximately one month; in other cases, a much longer time span exceeding 100 days was required, which in the laboratory and nature could be greater than the lifetime of a snail. Carbon and nitrogen reflected different time periods of diet in snail species. The half-life of δ^13^C (up to 6.4 days) was shorter than those of δ^15^N (up to 13.3 days) for *H*. *trivolvis* and *L*. *elodes*. In contrast, *L*. *stagnalis* δ^15^N (mean = 2.3 days) had a shorter half-life compared to δ^13^C (mean = 6.6 days).

**Fig 3:**
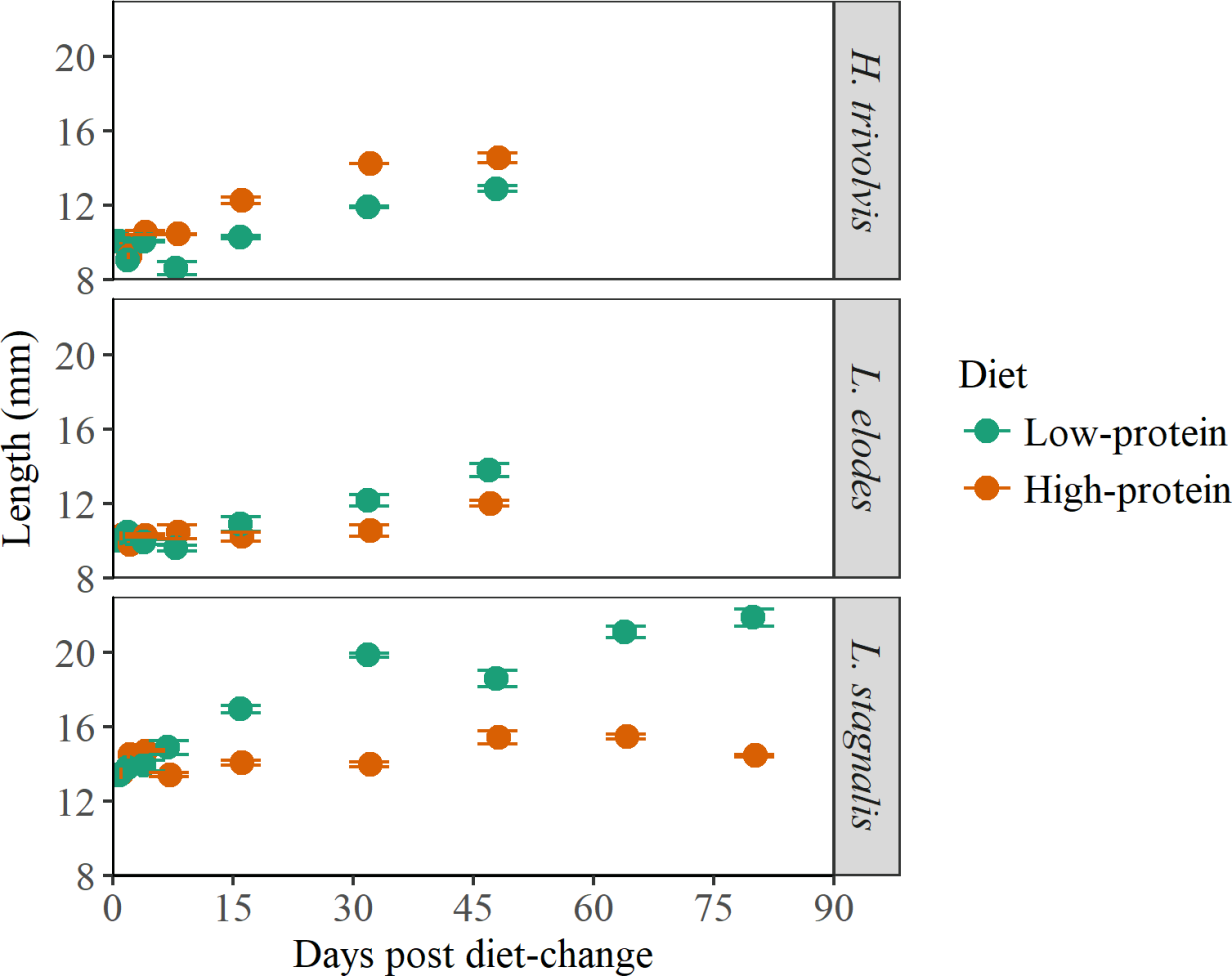
Shell length (mean ± SE) of three freshwater snail species fed a low-protein and high-protein diet.

Species differed in size (F_2,236_ = 721.96, *P* < 0.001), which could have occurred because diets were not switched for all species at exactly the same length. *Helisoma trivolvis* and *L*. *elodes* were on average ~10 mm, while *L*. *stagnalis* was ~14 mm when the diet was switched. However, the best model also included a significant species:day interaction (F_2,236_ = 12.02, *P* < 0.001) suggesting that differences in growth rate among the species varied over time (Fig 3). During the course of 48 days, *H*. *trivolvis* increased in growth (mean from two diets ± SE) by 37% (10.02 ± 0.13 to 13.72 ± 0.29 mm), while *L*. *elodes* and *L*. *stagnalis* increased by 27% (10.13 ± 0.16 to 12.88 ± 0.32 mm) and 26% (13.44 ± 0.20 mm to 17.00 ± 1.08 mm), respectively.

Diet quality affected size as a fixed factor (F_1,236_ = 6.14, *P* = 0.014) and in a 3-way interaction with species and day (F_3,236_ = 89.33, *P* < 0.001). This interaction indicates that after accounting for the fact that all snails would grow larger during the experiment, snail species were different sizes depending upon the diet. Most importantly, diet affected growth within a species; *Lymnaea* spp. grew larger on the low-protein diet compared to the high-protein diet while *H*. *trivolvis* grew larger on the high-protein diet compared to the low-protein diet (Fig 3).

### Diet-tissue discrimination factors

The best model for Δ^13^C had significant effects of diet and tissue and two interactions between species and diet, and food and tissue (r^2^ = 0.89). Assumptions of normality and heteroscedasticity were met. In the best model for Δ^15^N, species, diet and tissue were significant (r^2^ = 0.69). We used a log(Δ^15^N+2) to meet assumptions of normality and homoscedasticity.

Snails on different diets had significantly different Δ^13^C and Δ^15^N values (F_1_,_44_ = 92.69, *P* = < 0.0001; F_1_,_47_ = 73.15, *P* = < 0.001). Snails fed the high-protein diet had lower Δ^13^C and Δ^15^N values than those fed the low-protein diet regardless of tissue types (Tables 2 and 3). For snails fed the low-protein diet, all Δ^13^C and Δ^15^N values were positive (both tissues, all species, Tables 2 and 3). On the high-protein diet, snail Δ^13^C values were positive except for *L*. *elodes* (both tissues, Table 2), and Δ^15^N values were negative in gonad tissues for all three species, and in muscle of *H*. *trivolvis* (Table 3).

**Table 2:** δ^13^C values (‰, mean ± SD) and diet tissue discrimination factors (Δ^13^C) for freshwater snails (muscle and gonad) and their diets^a^.

		Low-protein diet			High-protein diet		
Species	Tissue	N	δ^13^C	Δ^13^C	N	δ^13^C	Δ^13^C
***H*. *trivolvis***	**Muscle**	21	-25.96 ± 0.32	2.47 ± 0.78	3	-18.79 ± 0.11	0.53 ± 0.11
***H*. *trivolvis***	**Gonad**	21	-27.46 ± 0.32	0.97 ± 0.78	3	-19.10 ± 0.10	0.22 ± 0.32
***H*. *trivolvis***	**Diet**	4	-28.43 ± 0.71	-	2	-19.32 ± 0.30	-
***L*. *elodes***	**Muscle**	20	-25.32 ± 0.53	2.52 ± 1.41	3	-20.10 ± 0.63	-0.66 ± 0.67
***L*. *elodes***	**Gonad**	20	-26.90 ± 0.56	0.94 ± 1.42	3	-20.06 ± 0.43	-0.62 ± 0.49
***L*. *elodes***	**Diet**	5	-27.84 ± 1.31	-	2	-19.44 ± 0.23	-
***L*. *stagnalis***	**Muscle**	24	-25.48 ± 0.30	2.44 ± 1.39	2	-18.32 ± 0.69	1.28 ± 0.70
***L*. *stagnalis***	**Gonad**	24	-27.10 ± 0.25	0.82 ± 1.38	2	-19.11 ± 0.25	0.49 ± 0.27
***L*. *stagnalis***	**Diet**	3	-27.92 ± 1.36	-	2	-19.60 ± 0.10	-

^**a**^Values for snails on the low-protein diet were calculated from all sample days, while values for snails on the high-protein diet were calculated 48 (*Helisoma trivolvis* and *Lymnaea elodes*) or 80 days (*Lymnaea stagnalis*) after the diet was switched.

**Table 3:** δ^15^N values (‰, mean ± SD) and diet tissue discrimination factors (Δ^15^N) for freshwater snails (muscle and gonad) and their diets^a^.

		Low-protein diet			High-protein diet		
Species	Tissue	N	δ^15^N	Δ^15^N	N	δ^15^N	Δ^15^N
***H*. *trivolvis***	**Muscle**	21	5.30 ± 0.42	1.85 ±1.81	3	8.81 ± 0.06	-0.05 ± 0.92
***H*. *trivolvis***	**Gonad**	21	3.66 ± 0.49	0.21 ± 1.83	3	7.64 ± 0.05	-1.22 ± 0.92
***H*. *trivolvis***	**Diet**	4	3.45 ± 1.76	-	2	8.86 ± 0.92	-
***L*. *elodes***	**Muscle**	20	5.62 ± 0.67	3.05 ± 2.20	3	9.24 ± 0.29	0.54 ± 0.47
***L*. *elodes***	**Gonad**	20	3.91 ± 0.85	1.34 ± 2.27	3	7.25 ± 0.55	-1.45 ± 0.66
***L*. *elodes***	**Diet**	5	2.57 ± 2.10	-	2	8.70 ± 0.37	-
***L*. *stagnalis***	**Muscle**	24	3.40 ± 1.99	5.28 ± 3.50	2	9.58 ± 0.10	1.15 ± 0.17
***L*. *stagnalis***	**Gonad**	24	1.37 ± 1.96	3.25 ± 3.48	2	7.95 ± 0.12	-0.48 ± 0.18
***L*. *stagnalis***	**Diet**	3	-1.88 ± 2.88	-	2	8.43 ± 0.14	-

^**a**^Values for snails on the low-protein diet were calculated from all sample days, while values for snails on the high-protein diet were calculated 48 (*Helisoma trivolvis* and *Lymnaea elodes*) or 80 days (*Lymnaea stagnalis*) after the diet was switched.

Tissue types differed in Δ^13^C (F_1,44_ = 215.37, *P* <0.0001) and Δ^15^N (F_1,47_ = 47.37, *P* <0.0001), and values for muscle were higher than gonad tissues (Tables 2 and 3). For Δ^13^C, there was also a significant interaction between diet and tissue (F_1,44_ = 15.83, *P* <0.0001). Tukey post-hoc tests found differences between the tissues on the low-protein diet (*P* < 0.002), but no differences between the tissues on the high protein (*P* > 0.10). On the low-protein diet, the average Δ^13^C ± SD for muscle was 2.48 ± 0.04‰, but the values for gonad on the low-protein diet, and gonad and muscle on the high-protein diet were more similar (0.91 ± 0.08‰, 0.38 ± 0.98‰ and 0.03 ± 0.58‰, respectively). For Δ^15^N, the average values for muscle and gonad tissue on the low-protein diet were 3.39 ± 1.74‰ and 1.60 ± 1.54‰, respectively; on the high-protein diet, the average Δ^13^C of muscle and gonad tissue were 0.55 ± 0.60‰ and -1.05 ± 0.51‰, respectively.

The model of Δ^13^C had significant interactions between species and diet (F_1,44_ = 9.31, *P* = 0.0004). Species had similar Δ^13^C on the low-protein diet and the high-protein diet (Tukey post-hoc tests with *P* ≥ 0.98), except for the significant difference between *Lymnaea* spp. fed high-protein (*P* = 0.001; -0.64 ± 0.03‰ (mean ± SD) for *L*. *elodes* and 0.89 ± 0.56‰ for *L*. *stagnalis*). For Δ^15^N, species differed (F_2,47_ = 28.81, *P* <0.0001). Tukey post-hoc tests showed that all pairwise differences between species were significant (*P* < 0.02) with mean ± SD of 0.20 ± 1.27‰, 0.87 ± 1.87‰ and 2.30 ± 2.51‰ for *H*. *trivolvis*, *L*. *elodes* and *L*. *stagnalis*, respectively.

## Discussion

Estimates of diet-tissue discrimination and isotopic turnover rates from controlled-feeding studies are essential for ensuring that stable isotope analysis accurately reflects wildlife diet and trophic relationships. Using freshwater snails, we found that diet quality affected DTDFs, which usually differed among species. Carbon turnover rates were similar among species for a given tissue type, but nitrogen turnover varied more among species. In addition, growth varied among species depending on diet; some species grew larger on high-protein (i.e. *H*. *trivolvis*) while others grew larger on low-protein diet (i.e. *Lymnaea* spp.). In turn, differences among species in growth influenced turnover rates; turnover was faster in the species with the highest growth rate when the diet was switched from low to high-protein. This result supports the hypothesis that growth is one of the main processes that affects tissue turnover [41]. Interestingly, this relationship was not true for DTDFs, where the pattern was the same for all species regardless of growth performance; DTDFs were always greater on low-protein than high-protein diets. This finding suggests that the same factor (i.e. diet quality) might influence two key parameters of stable isotope analysis differently.

Our Δ^13^C values were comparable to other studies of aquatic organisms. We observed a slightly smaller range of Δ^13^C values (-1.16 to +2.49‰) relative to the range (-2.1 to +2.8‰) reported for 14 aquatic invertebrates species that were sampled from freshwater and marine environments [42]. The mean Δ^13^C values from snails fed the low-protein diet (1.52‰) were similar to the mean of 1.33‰ for freshwater aquatic species (n = 42 studies of birds, fish and invertebrates) reported by Caut *et al*. [43]. This similarity in Δ^13^C suggests that if these values are unknown, the mean value could be used to predict the diet of freshwater aquatic organisms from stable isotope analysis.

Whether the same conclusions can be made about Δ^15^N is unclear. Post [2] recommended that animal studies should use 3.4‰, an average value derived from 56 studies that included a variety of animals, from copepods to polar bears, sampled in different environments (i.e. freshwater, marine and terrestrial). In our study, most Δ^15^N values were relatively smaller, with an overall average of 1.28‰, which indicates that using Post’s value could lead to inaccurate diet estimates depending upon the taxon. Yet estimates from previous studies on gastropods (-0.06‰) were quite similar to our estimates of snails on the high-protein diet (combined mean = 0.04‰), as δ^15^N of that diet was similar to the gastropod diet of those previous studies (high-protein diet) [44, 45]. This similarity suggests that mean Δ^15^N values from phylogenetically related species with similar diets may be useful for diet reconstruction using stable isotope analysis.

Snails fed diets with higher δ^13^C and δ^15^N values (i.e., high-protein diet) had lower DTDFs than those fed on diets with lower isotopic ratios (i.e., low-protein diet) (Table 1). This observation is consistent with other studies on a range of taxa including mammals, birds, fishes and invertebrates [43, 46]. Robbins *et al*. [8] predicted that Δ^15^N values should decrease as diet quality increases with trophic level, which has been observed in some types of consumers. For example, carnivores that ingested higher quality protein had lower Δ^15^N values than herbivores [8], and we confirmed that for the same consumer species, diets with higher quality had lower Δ^15^N values. Variation in DTDF by diet quality suggests that it is important to choose DTDFs generated by controlled-feeding studies that mimic diets in the wild. Otherwise, the diet estimates from stable isotope mixing models may not be accurate. These results suggest that different DTDFs should be applied to diet items that differ in protein content such as between animal prey and plants. This consideration could be important for omnivorous consumers that eat a mixture of animal and plant tissues that vary in protein content, and for consumers whose diet may be subject to changes in protein quality over space and time.

Although animal tissue is generally enriched in ^13^C compared to the diet [1], we observed negative Δ^13^C values for snails fed on the high-protein diet. Negative values could occur if carbohydrates with higher isotopic values than other dietary nutrition sources (e.g., protein) were mainly used as energy, instead of being incorporated into the tissue. In some controlled-feeding studies, mice were fed a combination of carbohydrate (cane sucrose) and protein (casein) that had distinctly different δ^13^C values [7, 18, 47]. Mice preferentially used protein to make new tissue components rather than carbohydrates, with roughly 75% of the carbon in the tissue being derived from protein [18]. Our high-protein diet had corn, which is a C_4_ plant with high δ^13^C values, potentially giving our high-protein diet a higher δ^13^C value. Negative Δ^13^C for snails on high-protein diet could also have occurred if most carbohydrate was lost as CO_2_ after being used as energy as hypothesized by Hobson and Stirling [48].

We also found that DTDFs and turnover rates differed among tissues. As predicted, more metabolically active tissue (i.e. gonad) tended to reflect the isotopic value of the diet more closely (mean Δ^13^C = 0.29‰ and Δ^15^N = 0.43‰) compared to muscle tissue (mean Δ^13^C = 1.35‰ and Δ^15^N = 2.13‰) and had shorter half-life (4.4 days) compared to muscle tissue (7.5 days). Differences among tissues types in DTDF and turnover rates are well-tested in vertebrates [6, 49], but most stable isotope studies of invertebrates used the whole organism [16, 44, 45]. Our study suggested that metabolic activity of tissue types could influence DTDFs and turnover rates in invertebrates. Thus, future studies on invertebrates should consider measuring stable isotope ratios of particular tissues rather than whole organisms and tissue-specific DTDFs just as studies of vertebrates do.

In general, freshwater snails had fast turnover rates, with half-life < 7 days for δ^13^C and < 14 days for δ^15^N. The quicker turnover rate for carbon was especially evident in the foot tissue of *L*. *elodes* and *H*. *trivolvis*. If diet reflects environmental change, then diet studies of snails could indicate environmental changes over short time scales in wetlands. Differences between these elements could occur because carbon is derived from both carbohydrates and proteins whereas nitrogen only comes from dietary protein [50]. The difference in turnover rates between carbon and nitrogen suggests that future studies could use different elements to better understand the temporal variation in ecosystems by estimating diet at different time periods. For example, δ^13^C values can be used to determine if recent diet differs, whereas δ^15^N values can be used to determine if diet differs over longer time periods. This approach could also be used to understand how diet changes during migration and differs among seasons.

Diet also affected snail growth. On the low-protein diet, all snail species had similar growth rates, but on the high-protein diet, some species grew larger (i.e. *H*. *trivolvis*) while others grew less (i.e. *L*. *elodes* and *L*. *stagnalis*). Furthermore, turnover rates for δ^13^C were faster on the diet for the species that achieved the greatest growth. For example, *H*. *trivolvis* had quicker turnover rates than *Lymnaea* spp. when snails were fed the high-protein diet.

Despite repeated calls for more controlled-feeding studies to estimate DTDFs and turnover rates, such studies remain infrequent but are essential for accurate use and interpretation of stable isotope models that estimate wildlife diet and trophic relationships. This study is the first to measure these two parameters for both carbon and nitrogen in freshwater gastropods, and our results suggest it is particularly important to estimate DTDF and turnover if diet quality may vary over space or time. If controlled-feeding studies using a diet that approximates the diet in the wild cannot be performed, Δ^13^C for freshwater animals is best predicted by using an average value from species living in a freshwater environment. In contrast, the larger variation in Δ^15^N suggests that it is best predicted from averaging values from several closely related species with similar diets. Moreover, our results suggest that as for vertebrates, tissue-specific values should be considered for invertebrates when estimating DTDF and turnover rates. The most significant finding from our study is that diet quality affected growth performance, which in turn affected DTDFs and turnover rates. This result has important implications for field studies of wildlife diet. In nature, the preferred diet may not always be available, suggesting that DTDFs and turnover rates may vary depending on the season and food availability.
